# Unusual presentation of lung carcinoma with pituitary metastasis: a challenging diagnosis and sodium management dilemmas

**DOI:** 10.1530/EO-22-0064

**Published:** 2022-09-06

**Authors:** Poh Shean Wong, Subashini Rajoo, Hairuddin Achmad Sankala, Mohamed Badrulnizam Long Bidin

**Affiliations:** 1 Endocrine Unit, Medical Department, Hospital Kuala Lumpur, Malaysia; 2 Radiology Department, Hospital Kuala Lumpur, Malaysia

## Abstract

**Summary:**

Pituitary metastasis (PM) is a rare complication of an advanced malignancy. Albeit rare, PM can be more detected and achieve a longer survival rate through frequent neuroimaging and newer oncology therapies. Lung cancer is the most frequent primary site, followed by breast and kidney cancers. Patients with lung cancer generally present with respiratory symptoms and are commonly diagnosed at an advanced stage already. Nevertheless, physicians should be mindful of other systemic manifestations as well as signs and symptoms related to metastatic spread and paraneoplastic syndromes. Herein, we report the case of a 53-year-old woman who presented with PM as the first sign of an undiagnosed lung cancer. Initially, her condition was a challenging diagnosis and was even complicated with diabetes insipidus (DI), which can present as severe hyponatremia when coexisting with adrenal insufficiency. This case also highlights that treating DI with antidiuretic hormone (ADH) replacement was complicated by extreme difficulties in attaining satisfactory sodium and water balance during the clinical course, with the possibility of coexistent DI and syndrome of inappropriate ADH secretion because of the underlying lung cancer.

**Learning points:**

- When patients present with pituitary mass and diabetes insipidus (DI), pituitary metastasis should be considered as an initial differential diagnosis. DI caused by pituitary adenoma is rare and is typically a late finding.
- DI can present as severe hyponatremia when coexisting with adrenal insufficiency.
- Cortisol can directly inhibit endogenous antidiuretic hormone (ADH) secretion. Patients with adrenocorticotropic hormone deficiency will have increased tonic ADH activity and subsequently reduced capacity for free-water excretion. However, when on steroid therapy, patients should be monitored for possible DI because steroids can restore free-water excretion.
- A substantial change in serum sodium after desmopressin treatment should eliminate the possibility of desmopressin overdose or coexistence of DI and syndrome of inappropriate ADH secretion in patients with lung cancer. Therefore, frequent monitoring of serum sodium concentrations is crucial.

## Background

The incidence of lung cancer is increasing worldwide, particularly in developing countries. In the Asia-Pacific region, lung cancer is the most commonly diagnosed cancer among men and the second most common cancer among women after breast cancer. In 2018, 1.2 million people were diagnosed with lung cancer, and it was the leading cause of cancer-related deaths, with slightly over 1 million deaths in the region (OECD/World Health Organization 2020). In particular, lung cancer is one of the most common causes of cancer-related deaths in Malaysia (Ministry of Health Malaysia 2021). Early diagnosis is crucial to improve the overall survival of patients with lung cancer. These patients generally present with respiratory symptoms and are commonly diagnosed at advanced stage already. Nevertheless, physicians should be mindful of other systemic manifestations related to metastatic spread and paraneoplastic syndromes. Lung carcinoma metastasizes most commonly to the liver, adrenal glands, bones, respiratory system, and brain parenchyma (Riihimäki *et al.* 2014). The pituitary gland is an unusual location for metastasis (Habu *et al.* 2015)*.* In this case presentation and literature review, we aimed to discuss a case of pituitary metastasis (PM) as the first sign of an undiagnosed malignancy complicated with diabetes insipidus (DI).

In addition, the treatment of DI with antidiuretic hormone (ADH) replacement was complicated by extreme difficulties in attaining satisfactory sodium and water balance during the clinical course, with the possibility that DI and syndrome of inappropriate antidiuretic hormone secretion (SIADH) can coexist because of the underlying lung cancer.

## Case presentation

**Figure 1:**
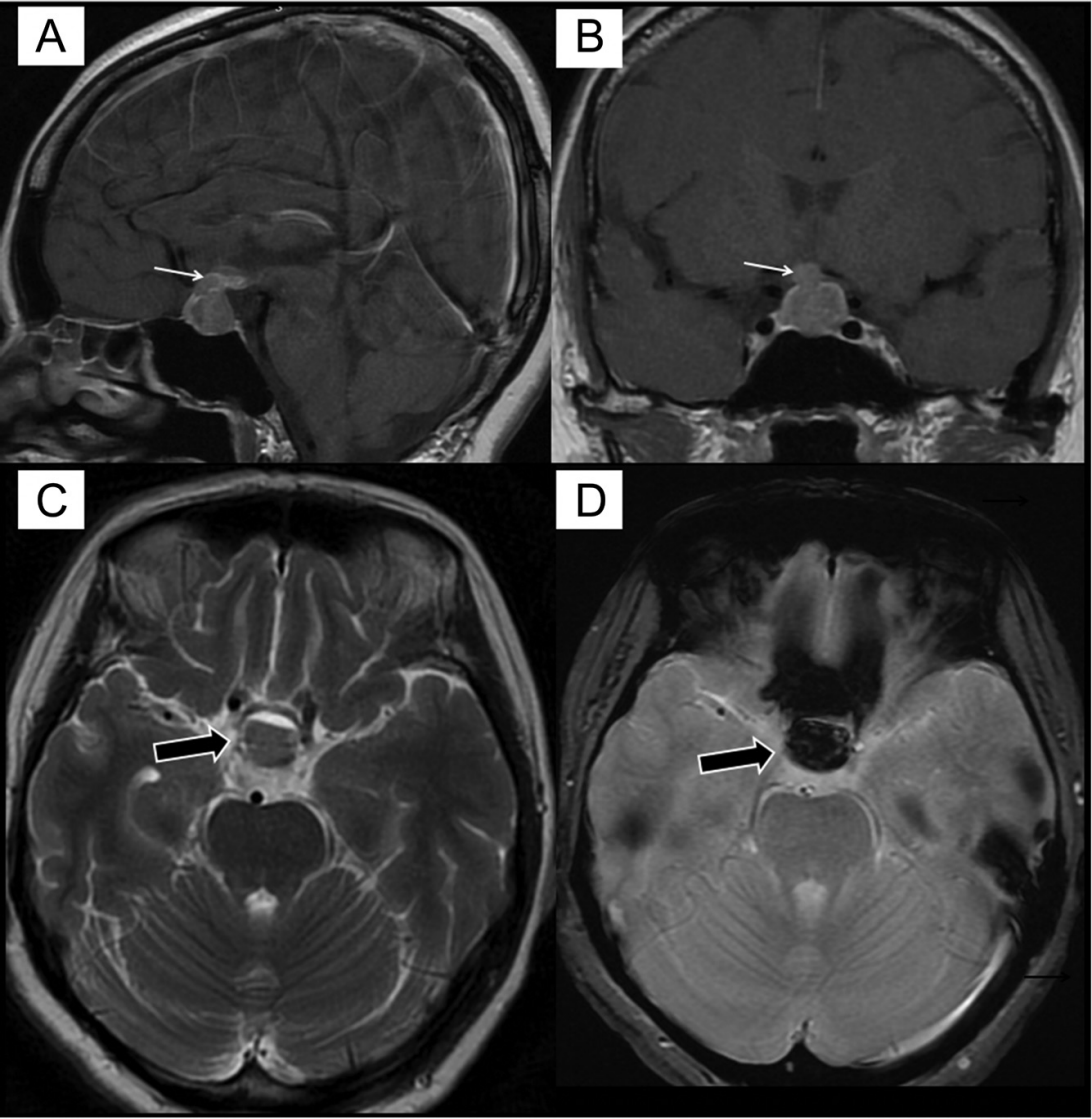
Sagittal (A) and coronal (B) sections of contrast-enhanced T1W images showing sellar mass with suprasellar extension and stalk involvement (white arrows). Axial T2W image (C) showing fluid–fluid level (black arrow) within the lesion. Axial gradient echo (GRE) image (D) showing blooming artifact (black arrow) within the lesion indicating hemorrhage.

A 53-year-old woman with no known medical illness presented with bilateral peripheral vision loss. She had no headache, vomiting, or hypo- or hyperpituitary functioning symptoms. Visual field assessment showed bitemporal hemianopia. MRI of the brain showed a contrast enhancing hemorrhagic pituitary mass with suprasellar extension, measuring 1.7 × 1.9 × 2.0 cm (anteroposterior × W × craniocaudal). The pituitary stalk was also involved, causing thickening (Fig. 1A and B). The patient refused to undergo transphenoidal surgery. Thus, she was treated conservatively and was not required to receive any hormone replacement then. Two months later, she presented with worsening headache and vision, polyuria, and polydipsia. Hence, she was treated for pituitary macroadenoma with apoplexy and was started on i.v. hydrocortisone. Additionally, she developed DI and secondary hypothyroidism; thus, desmopressin (0.1 mg tablet) once a day and thyroxine (25 mcg tablet) were administered once a day. Subsequently, she was counseled again for operation.

## Investigation

Eventually, patient agreed and underwent endoscopic transphenoidal surgery. Histopathological examination revealed pituitary metastatic adenocarcinoma. Immunohistochemistry staining showed strong positivity for CK7, CKAE1/ AE3, and EMA and negativity for CK20, ER, PR, mammoglobin, vimentin, and GFAT. Thereafter, she experienced left upper limb pain caused by fracture of the neck of left humerus. CT scan of the thorax, abdomen, and pelvis showed a left lower lobe mass (3.3 × 4.9 × 3.6 cm) with multiple bilateral lung nodules, left humerus lytic lesion, and vertebral body lytic lesion at L5. When she had worsened high intracranial pressure symptoms, a ventriculoperitoneal shunt was inserted, and CT scan revealed hydrocephalus and cerebral edema. A biopsy of the left lung mass showed pulmonary adenocarcinoma, positive for EGFR, TTF1, and CK7. The final diagnosis was advanced lung adenocarcinoma with metastases to the lung, bone, and pituitary gland.

## Treatment

She received whole-brain radiotherapy, left humerus radiotherapy, and oral gefitinib (250 mg) therapy once a day. She was also on hormonal supplement, namely, thyroxine (25 mcg tablet) once a day, desmopressin (0.1 mg tablet) once a day, and hydrocortisone (10 mg tablet) twice a day. Immediate surveillance scans showed that the left lung mass and pituitary mass shrank. Her vision also improved. Unfortunately, she did not comply with gefitinib therapy because of adverse cutaneous reactions. A skin biopsy showed cutaneous metastasis secondary to lung carcinoma. Further, PET scan was suggestive of fluorodeoxyglucose (FDG)-avid cutaneous metastasis; however, mild FDG avidity in the left lung nodule and lytic lesion at the left humerus was indeterminate. Fortunately, the patient tolerated gefitinib at a lower dose (250 mg tablet) administered every other day. However, after 2 years, scans showed disease progression.

## Outcome and follow-up

After disease progression, patient defaulted her follow-up and treatment. Few months later, she presented with lethargy, vomiting, and giddiness. Clinically, she was dehydrated and lethargic, with a full Glasgow coma scale. Her serum sodium level upon admission was 118 mmol/L (Table 1). Hence, i.v. hydration and i.v. dexamethasone were started. The next day, her sodium rose substantially to 143 mmol/L, but she became polyuric, with a urine output of 3.6 L. On the following day, her sodium level, serum osmolality, and urine osmolality were 155 mmol/L, 327 mOsm/kg, and 88 mOsm/kg, respectively. Her calcium and glucose levels remained within the normal range. She was then treated for DI with subcutaneous desmopressin.

**Table 1:** Serial blood tests, fluid balances, and treatments during patient’s hospitalization.

Day	1	2	3	4	4	4	5	5	6	6	7	8	9	10	11
Serum sodium (mmol/L)	118	143	155	152	131	125	121	122	131	136	135	135	136	141	137
Serum osmolality (mosm/kg)	244		327			252	252		270	289	285	278	284	286	
Urine osmolality (mosm/kg)	104		88	44		627	640		179	99	99	54	74	42	
Urine sodium (mEq/L)	41		17	19		236	247			37	25				
Fluid balance (mL)	200	−1900	−300			700		−2600		−200	−600	−10	−150	150	1000
Treatment															
Dexamethasone	i.v. 8 mg BID	i.v. 8 mg BID	i.v. 8 mg OD	i.v. 8 mg OD			T 4 mg OD		T 4 mg OD		T 4 mg OD	T 2 mg OD	T 2 mg OD	T 2 mg OD	T 2 mg OD
Desmopressin			s.c. 2 mcg	s.c. 1 mcg						T 0.05 mg	T 0.05 mg	T 0.05 mg	T 0.05 mg	T 0.05 mg	T 0.05 mg
Radiotherapy (day)							1		2		3	4	5	6	7

BID, twice a day; OD, once a day; T, tablet.

Repeated scans of the brain, thorax, abdomen, and pelvis showed an empty sella, a mildly thickened remnant of the infundibular stalk, and an increased number of lung masses and nodules. We also found metastases to multiple bones with extraosseous soft-tissue components, causing spinal canal narrowing, which was worst at T6 and T9 vertebral levels, with a pathological compression fracture.

However, hyponatremia developed approximately 12 h after the second dose of desmopressin and persisted after discontinuing desmopressin (Table 1). It was only resolved after few days, with the lowest at 121 mmol/L. The patient became symptomatic; thus, hypertonic saline 3% therapy was started. Concurrently, she underwent spinal radiotherapy. Hyponatremia was then resolved, and the sodium level has remained stable. Subsequently, she required a daily dose of desmopressin (0.05 mg tablet) once a day. Eventually, she was discharged without untoward events. Considering her disease progression, oral tyrosine kinase inhibitor was switched to i.v. chemotherapy.

## Discussion

PM is a rare complication of an advanced malignancy. However, owing to the greater availability of neuroimaging modalities and advancement of oncological therapies, PM has already been discovered more frequently in patients with disseminated cancer. Unfortunately, it seldom presents as the first sign of malignancy, as in our case. Most cases are asymptomatic and are incidentally discovered during an autopsy or in patients during the end-stage malignant course, and characteristic symptoms are reported in <20% (Komninos *et al.* 2004).

Lung carcinoma metastasizes most commonly to the liver, adrenal glands, bones, and brain parenchyma, and rarely to unusual locations (Riihimäki *et al.* 2014). The pituitary gland is one of the unusual locations for metastasis, accounting for approximately 0.4% of all intracranial metastatic tumors (Habu *et al.* 2015) and representing less than 1% of surgically treated pituitary tumors (Fassett & Couldwell 2004). According to a recent systemic review of PM, the lungs are the most frequent primary site of cancer, followed by breast and kidney cancers (Ng *et al.* 2020). However, when patients with primary lung cancer present without respiratory symptoms, the diagnosis is frequently delayed because of the unusual presentation of pituitary symptoms, as in our patient. Our case demonstrated symptomatic PM as the first presentation of an undiagnosed malignancy, which was initially thought to be a symptomatic benign pituitary macroadenoma. Based on the literature review, which involved 289 PMs reported from 1957 to 2018, visual problems are the most common first manifestation of PM (48.8%), followed by DI (38.4%), panhypopituitarism (37.7%), and headache (35.3%) (Javanbakht *et al.* 2018). Our patient experienced all of these manifestations. According to a meta-analysis, DI is the most common clinical manifestation of PM (He *et al.* 2015). Clinical presentation may help differentiate PM from pituitary adenoma, as in the case of our patient. When a patient first presents with pituitary mass and DI, PM should be considered as the initial differential diagnosis (Morita *et al.* 1998). DI caused by pituitary adenoma is typically a late finding, with a rare incidence of 1% (Sioutos *et al.* 1996). The reason is that metastases target the posterior lobe of the pituitary gland and the infundibulum rather than the anterior lobe, likely because of the hematological spread via the neurohypophyseal blood vessels (Komninos *et al.* 2004). Other features that may lead to PM include an enhancing, rapidly growing suprasellar mass with signs of neurological or other hormonal impairments in patients above 50 years old (Famini *et al*. 2011).

In a systemic review of 657 PM cases, the median survival was 14.0 months, with lung cancer tending to have a shorter survival rate (9 months), compared with 22 months in breast cancer and 30 months in kidney cancer (Ng *et al.* 2020). Results from this systemic review emphasized the central role of radiotherapy, which has shown to significantly improve patients’ survival rate (Ng *et al.* 2020). The authors concluded that the management of patients with PM, especially in those with breast and kidney carcinoma, should not be limited to terminal-stage care. Furthermore, the indication for pituitary surgical intervention upon diagnosis should be restricted to patients with acute ophthalmological involvement and those who require histopathological biopsy in case of unknown or doubtful primary cancer. Our patient underwent transphenoidal surgery, which improved her vision, and was diagnosed with a pituitary lesion. Overall survival was significantly longer in patients receiving local radiotherapy and chemotherapy. Multidisciplinary discussion is crucial for the control of endocrinopathies and the addition of systemic oncological treatments tailored for primary histology (Ng *et al.* 2020). Our patient underwent transsphenoidal surgery and radiotherapy and was treated with oral tyrosine kinase inhibitor for 3 years before disease progression; her survival period was longer than that of patients with lung cancer (9 months) observed in a recent systemic review (Ng *et al.* 2020). Therefore, PM management should not be confined to terminal-stage care.

Another unique aspect of the present case was that after the patient had defaulted her hormonal replacement for few months, her serum sodium concentrations remarkably changed. Hypovolemic hyponatremia secondary to dehydration was considered upon initial presentation, and i.v. hydration was initiated. Concurrently, i.v. dexamethasone was started to treat for possible cerebral edema owing to brain metastasis. Notably, her severe hyponatremia improved markedly on the second day. However, she developed polyuria, hypernatremia, high plasma osmolality, and low urine osmolality, which responded with desmopressin administration. These findings indicated central DI secondary to PM, which was unmasked by corticosteroid therapy. Thus, DI can present as severe hyponatremia when coexisting with secondary adrenal insufficiency. Watanabe *et al*. illustrated high-grade hyponatremia in a patient with lung cancer manifesting PM, with the coexistence of adrenal insufficiency and DI (Watanabe *et al.* 2020). Cortisol can directly inhibit endogenous ADH secretion. Hence, patients with adrenocorticotropic hormone deficiency will have increased tonic ADH activity and subsequently reduced capacity for free-water excretion. When their patient had steroid accumulation, the ability to excrete free water was restored (Huang *et al.* 2005, Ohta *et al.* 2006). Therefore, in our patient, classical features of DI developed soon after commencing dexamethasone.

Interestingly, after desmopressin therapy was started, the serum sodium and osmolality levels decreased substantially, requiring hypertonic saline correction. This phenomenon was initially thought to be caused by desmopressin overtreatment, but her hyponatremia persisted after desmopressin discontinuation and only resolved after few days, more than the presumed clearance of desmopressin. The possible explanation could be the concurrent paraneoplastic secretion of ADH from her lung cancer, leading to SIADH euvolemic hyponatremia. Desmopressin overdose was unlikely because her hyponatremia lasted beyond the expected clearance of desmopressin. However, the association of SIADH with non-small cell lung cancer (NSCLC) has been reported by very few studies. Our case is unique in that after radiotherapy, the patient’s SIADH improved shortly, and her serum sodium level stabilized. Ishihara *et al*. also reported SIADH improvement in a patient with NSCLC who underwent radiotherapy without chemotherapy (Ishihara *et al.* 2019).

Our center has no facility to run ADH concentration, but biochemical investigations taken during her hyponatremic episodes were suggestive of SIADH. Instead of fluid restriction, the patient was allowed to drink when feeling thirsty, owing to recent DI and the risk of dehydration and hypernatremia recurrence. The patient subsequently underwent spinal radiotherapy. Hyponatremia was then resolved, and the sodium level stabilized. Eventually, the patient was discharged well.

In conclusion, we present a rare case of advanced lung carcinoma with PM, complicated with cranial DI. PM should be considered in older patients presenting with new-onset DI with or without anterior pituitary dysfunction. Early recognition will help in the timely initiation of therapies and maximizing patient’s quality of life. The case also demonstrated challenging management dilemmas regarding the patient’s serum sodium concentration, which give rise to several interesting aspects of sodium homeostasis, including the corticosteroid effect on ADH and the possibility of concomitant DI and SIADH. Importantly, physicians should be aware that DI can present as severe hyponatremia when coexisting with secondary adrenal insufficiency.

## Declaration of interest

The authors declare that there is no conflict of interest that could be perceived as prejudicing the impartiality of this case report.

## Funding

This work did not receive any specific grant from any funding agency in the public, commercial, or not-for-profit sector.

## Patient consent

Written informed consent has been obtained from patient for publication of the submitted article and accompanying images.

## Author contribution statement

All authors have contributed substantially in drafting and revising the case report, acquisition, analysis and interpretation of data for case report.
